# Intense laser interaction with micro-bars

**DOI:** 10.1038/s41598-023-48866-z

**Published:** 2023-12-04

**Authors:** Michal Elkind, Itamar Cohen, David Blackman, Talia Meir, Lior Perelmutter, Tomer Catabi, Assaf Levanon, Siegfried H. Glenzer, Alexey V. Arefiev, Ishay Pomerantz

**Affiliations:** 1 The School of Physics and Astronomy, Tel Aviv University, 69978 Tel Aviv, Israel; 2 Center for Light-Matter Interaction, Tel Aviv University, 69978 Tel Aviv, Israel; 3 Department of Mechanical and Aerospace Engineering, University of California San Diego, La Jolla, CA 92093 USA; 4 The School of Electrical Engineering, Tel Aviv University, 69978 Tel Aviv, Israel; 5 SLAC National Accelerator Laboratory, Menlo Park, CA 94025 USA

**Keywords:** Plasma-based accelerators, Nanophotonics and plasmonics

## Abstract

Intense laser fields interact very differently with micrometric rough surfaces than with flat objects. The interaction features high laser energy absorption and increased emission of MeV electrons, ions, and of hard x-rays. In this work, we irradiated isolated, translationally-symmetric objects in the form of micrometric Au bars. The interaction resulted in the emission of two forward-directed electron jets having a small opening angle, a narrow energy spread in the MeV range, and a positive angle to energy correlation. Our numerical simulations show that following ionization, those electrons that are pulled into vacuum near the object’s edge, remain in-phase with the laser pulse for long enough so that the Lorentz force they experience drive them around the object’s edge. After these electrons pass the object, they form attosecond duration bunches and interact with the laser field over large distances in vacuum in confined volumes that trap and accelerate them within a narrow range of momentum. The selectivity in energy of the interaction, its directionality, and the preservation of the attosecond duration of the electron bunches over large distances, offer new means for designing future laser-based light sources.

The interaction of intense laser fields with matter at its sharp interface with the vacuum is central to understanding phenomena like energy absorption of intense light in matter^1^, emission of coherent soft X-ray radiation from irradiated polished surfaces^2^, and formation of sheath fields responsible for MeV ion emission from irradiated thin foils^3^. Following ionization, the laser fields near the interface, which are enhanced by diffraction, extract electrons from the plasma^4^. At these intensities, the magnetic and electric terms of the Lorentz force become comparable, and the so-called $J\times B$ heating mechanism participates in transferring laser energy to the electrons^5^.

After half an optical cycle as the laser field changes direction, these electrons would be pushed back. If the irradiated object is sufficiently large, the electron trajectories are likely to drive them back into the plasma, where their energy will be dissipated through collisions. This is the basis for the vacuum heating (VH) model^1^ which predicts the energy transfer of laser light to flat surfaces and its sensitivity to the laser’s polarization and angle of incidence^6^.

In the case of *rough* surfaces, a range of experiments have shown diverse emission features when the irradiated objects possessed geometric structures with dimensions on the order of the laser wavelength or smaller. These include the irradiation of surfaces coated with nanowires^7–10^, micrometric scale pillars^11–13^, plastic spheres^14–16^, water droplets^17^, and modulated “grating” targets irradiated at incidence angles close to the resonant condition for surface plasmon excitation^18,19^. Compared to the irradiation of flat surfaces, these objects present higher ionization level^10^, increased laser absorption^8,16^, volumetric heating into ultrahot plasma^20^ and high-energy X-ray emission^7,13,14,17^, as well as the emission of MeV electrons^11,13^, protons^12,15,16^, and fusion neutrons^9^.

Proposed explanations for these features include local enhancement of the electromagnetic fields^11,12^, multi-pass stochastic heating of electrons through Mie resonance^14^, and an increased number of possible trajectories that enable electrons to undergo efficient VH^13,15^.

In those experiments, the stochastic nature of the targets’ geometric features along with the typical laser’s pointing instability, raise substantial uncertainty about the illuminated structure details.

Clearly *isolated* micrometric targets with well-defined geometries better serve the purpose of understanding the reaction mechanism. In the particular case of targets which are circularly symmetric in the laser’s polarization plane, the interaction is readily analyzed in the framework of Mie theory^21^. One such numerical investigation for the case of irradiated He droplets^4^ revealed that enhancement of the local field at the target surface ejects electrons with MeV-level energies into vacuum. The interaction results in two jets comprised of attosecond-duration electron bunches emitted at certain angles set by the ratio of the droplet radius to the wavelength and the ratio of the plasma frequency to the laser frequency. The authors noted that the angular distribution of the emitted ions follows the predicted Mie angles better than that of the electrons. They attributed this feature to modifications in the electrons direction as they move away from the droplet. These modifications will be discussed in the work presented here.

The emission of these two electron jets was first observed experimentally by Cardenas et al.^22^ who irradiated the tips of W needles with laser intensities in the range of $a_0$ = 0.2–5.3, where $a_0$ is the normalized laser intensity which for a laser pulse of wavelength $\lambda$ is given in practical units by $a_0$ = $0.86\lambda \ [\mu m]\ \sqrt{I [10^{18}\ \mathrm {Wcm^{-2}}]}$. Unstable laser pointing combined with the 3D geometry of the conically-shaped targets resulted in an unknown effective irradiation area^23^. Nevertheless, under the assumption that Mie scattering governs the emission, the authors were able to infer the effective target radius of each shot from the opening angle between the electron jets, and found it to be insensitive to the laser intensity. Mie scattering would not account for the observed multi-MeV electron energies, which the authors attributed to subsequent interaction with the laser fields in vacuum^24^.

**Figure 1:**
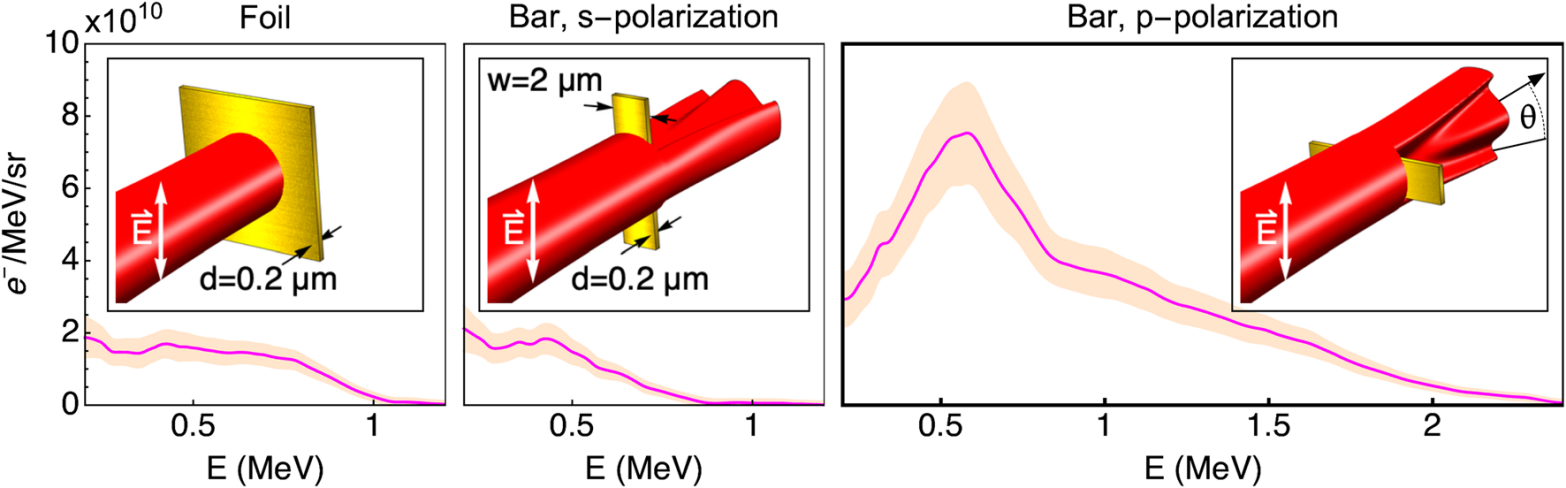
Measured spectra of electrons emitted from d = 0.2 $\upmu$m thick Au targets under irradiation with a relativistic laser pulse ($a_0$ = 2.7) in three configurations: a flat foil which is much wider than the focal spot size; a *w* = 2.0 $\upmu$m wide bar in s-polarization (bar is parallel to $\vec {E}$); and a bar with the same dimensions irradiated in p-polarization.

To understand how intense light couples to wavelength-scale formations, we irradiated (see Methods) single micrometric Au bars (”micro-bars”) with a rectangular cross-section of (*w* = 2.0–6.0 $\upmu$m, perpendicular to the beam direction) x (*d* = 0.2 $\upmu$m, along the beam direction), which were completely immersed in the focal volume of an intense laser field, as illustrated in Fig. 1. The figure shows a comparison of the measured energy spectra of electrons emitted from these micro-bars, along the laser direction, when positioned perpendicular (”p-polarization”) or parallel (”s-polarization”) to the laser’s electric field, as well as for irradiation of an Au foil of the same thickness. The increased yields and coupling of laser energy to high-energy electrons is clearly observed in the case of a micro-bar irradiated in p-polarization.

**Figure 2:**
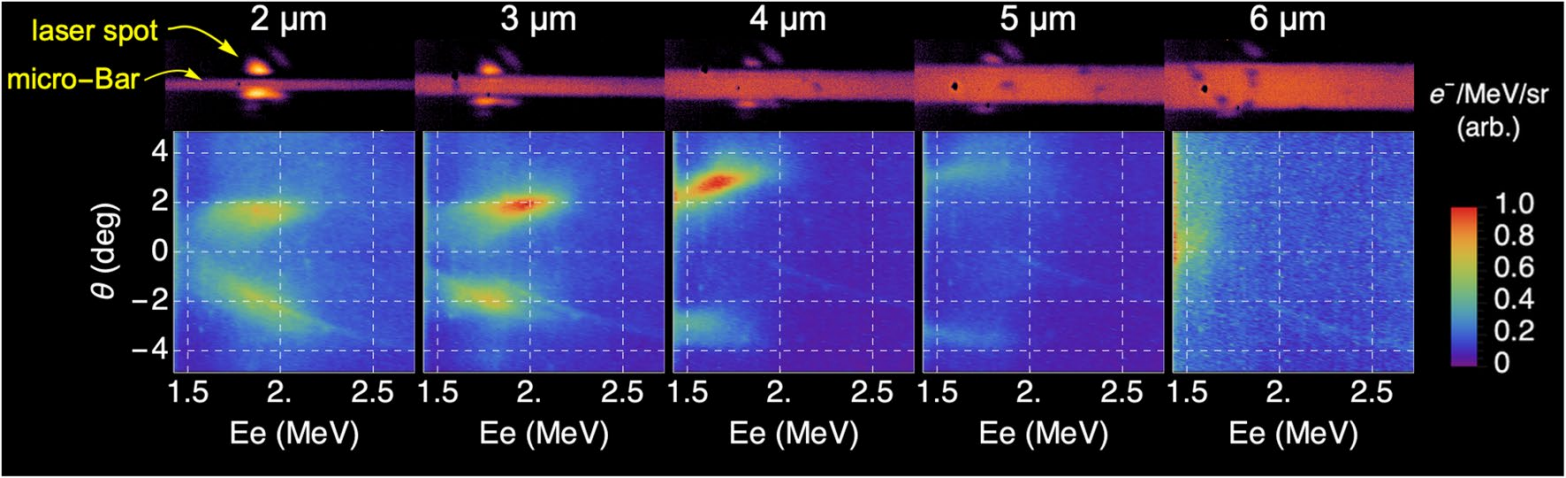
Measured angle-resolved spectra of electrons emitted from Au micro-bars, irradiated with an intense laser pulse ($a_0$ = 2.7) in p-polarization. The angle $\theta$ is defined to be in the polarization plane of the electric field, as indicated in Fig. 1. The micro-bars are all d = 0.2 $\upmu$m thick, and their width, in the range of *w* = 2–6 $\upmu$m, is indicated on top. Also shown are microscope images of the micro-bars, with 10x magnification, featuring the laser spot in low power prior to irradiation.

Figure 2 shows angle-resolved energy spectra of electrons emitted from *w* = 2–6 $$\upmu $$m wide micro-bars, following irradiation by a p-polarized, $$a_0$$ = 2.7 laser pulse. Microscope images of each of the micro-bars prior to irradiation, which also feature the laser spot in low power, are shown for each case. Two lobes of high-energy electrons, with a low separation angle ($$<4^\circ $$) are observed around the bar’s symmetry axis. These electrons are confined to a narrow energy range and present a positive angle-to-energy correlation. As the width of the micro-bar approaches the focal spot size, the emission angle increases while the number of electrons falls. The shot-to-shot variability in the relative intensity of the two jets |I$$_{Left}$$-I$$_{Right}|$$/(I$$_{Left}$$+I$$_{Right})$$ is found to have a mean value of 9.5%. This variability is attributed to the laser’s pointing instability, which was measured to be 0.43 $$\upmu $$m (RMS). We note that the same asymmetry between the two electron lobes can also arise from the random phase of the sub-cycle emission process when using *few-cycle* laser pulses^22^.

The underlying dynamics was revealed through particle-in-cell (PIC) simulations using the EPOCH code^25^ (see the Methods section for details). Figure 3a shows a snapshot from a 3D PIC simulation for a *w* = 2 $\upmu$m wide micro-bar irradiated with a p-polarized $a_0$ = 4.6 laser pulse. The snapshot was taken at t = 0, when the peak of the laser field impinged on the micro-bar, i.e., time of maximum electron emission. The transverse component of the laser field is shown in a red-to-blue color scale. Superimposing the emitted electrons on Fig. 3a, with their energy density distribution shown in green, clearly shows a double-jet formation comprised of attosecond electron bunches spanning over about four optical cycles.

**Figure 3:**
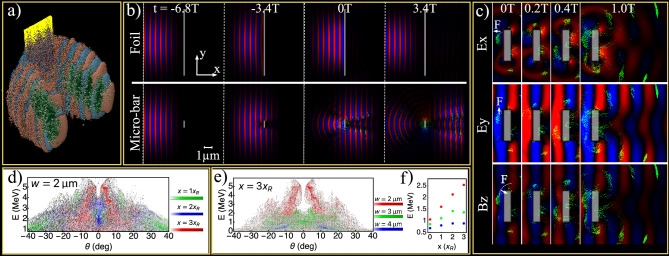
PIC simulation results. (**a**) A snapshot from a 3D-PIC simulation taken at t = 0, when the peak of a p-polarized $a_0$ = 4.6 laser field, impinges on the micro-bar. The transverse component of the electric field ($E_y$) is shown in a red-to-blue color scale. The density of electrons having energy above a threshold of 1 MeV is shown in green. (**b**) Four snapshots of two 2D-PIC simulations for the irradiation of 0.2 $\upmu$m thick targets: a *w* = 10.0 $\upmu$m wide foil (top) and a *w* = 1.0 $\upmu$m wide micro-bar (bottom). Both simulations ran in otherwise identical conditions, and the color scales are the same as in (**a**). (**c**) Four snapshots from a 2D-PIC simulation taken over one optical cycle at $0< t < 1\textrm{T}$. The blue-to-red color scales indicate the field strength of the electric field in the longitudinal ($E_x$) and transverse ($E_y$) directions, and of the out-of-plane magnetic field ($B_z$), each at a different arbitrary self-normalized scale. The Lorentz force exerted by each of the field components on one example electron bunch is indicated by white arrows. (**d**) Three snapshots of angle-resolved electron energy spectra for irradiation of a *w* = 2.0 $\upmu$m wide micro-bar, taken after a propagation distance of 1 (green), 2 (blue), and 3 (red) Rayleigh ranges. (**e**) Three snapshots of angle-resolved electron energy spectra taken after a propagation distance of 3$x_R$, for the cases of *w* = 2.0 $\upmu$m (red), *w* = 3.0 $\upmu$m (blue), and *w* = 4.0 $\upmu$m (green) wide micro-bars. The PIC simulations in (**d**) and (**e**) were performed with the actual laser intensity of the experiments ($a_0$ = 2.7). (**f**) Average electron energy at t = 0, and after propagation of 1, 2, and 3 Rayleigh ranges.

A confirmation that indeed the emission of high-energy electrons is a consequence of the micrometric dimensions of the micro-bar is given in Fig. 3b, in which 2D PIC results for an extended target (top) and a *w* = 1.0 $\upmu$m wide micro-bar (bottom) in otherwise identical conditions are compared. The density of high-energy electrons (> 1 MeV) is shown in green, and is observed only for the micro-bar case. See Supplementary Video 1 for the full animation of this simulation.

The origin for these high-energy electron bunches is identified by following the Lorentz forces they experience. Figure 3c shows four simulation snapshots taken within the duration of a single optical cycle following the peak of the interaction, under the same conditions as in Fig. 3b. The longitudinal (E$_x$) and transverse (E$_y$) components of the electric field and the out-of-plane magnetic field (B$_z$) are shown with red-to-blue color scales. The density of high-energy (E > 1 MeV) electrons is shown in green. Due to diffraction, the field amplitudes close to the target edge reach values up to 2.1 times higher than the laser’s field. The forces exerted by each of the field components on one electron bunch are indicated with white arrows. E$_x$ pulls and pushes electrons within each half optical cycle, in synchronization with E$_y$, which accelerates them in alternating directions. Therefore at each half cycle, electrons emerging close to one of the edges of the micro-bar are pushed in a trajectory beyond the extent of the target. Between t = 0 and t = 0.4T, the relativistic electron bunch overlaps with the same half-optical-cycle. Thus, the out-of-plane magnetic field introduces a $v \times B$ motion which rotates the bunch around the object’s corner. In the VH model for extended objects, the transverse motion of the electrons has no effect on the energy absorption, and the analysis is often conducted in a reference frame boosted in the transverse direction, so that only a 1D longitudinal motion remains^2^. Since the electron emission is in the electric field polarization plane^4^, the same description also applies to electrons emitted from a micro-bar irradiated in s-polarization (Fig. 1, middle), where the electron trajectories are parallel to the long dimension of the micro-bar. But in p-polarization, when the target posses micrometric features, the combined effect of E$_y$ and B$_z$ may push electrons around the target. This is the origin of the two-lobe formation observed in the experiment.

We now turn to the interaction of the bunched electrons with the diffracted laser fields while propagating over a long distance in vacuum. Three snapshots of angle-resolved electron spectra are shown in Fig. 3d, taken after propagation distances of 1 (green), 2 (blue), and 3 (red) Rayleigh ranges ($x_R$). Even at these large distances, some electrons continue to accelerate while maintaining a low divergence angle. At 3$x_R$, the highest energy electrons (E > 5 MeV) present a positive angle-to-energy correlation, as observed in the experiment. Another experimental observation that is recreated in the PIC (Fig. 3e) concerns the diminishing electron energies and the increase in the emission angle for wider micro-bars. The overall higher electron energies observed in the simulations compared to the experiment, are typical to 2D numeric effects^26^.

The average energy of the electron bunches at their peak emission time, and following their propagation over 1, 2, and 3 Rayleigh ranges is plotted in Fig. 3f, with the same color coding of Fig. 3e. At the time of emission, the electron energy is found to decrease linearly with *w*, i.e. with the square root of the focal intensity distribution. This is consistent with the energy scaling of electrons oscillating in the transverse laser field^27^ typically observed for intensities above 10$^{18}$ Wcm$^{-2}$. Due to the reduction of the transmitted laser fields, the average energy of electrons emitted from a *w* = 4 $\mu$m bar is increased by only 30% after propagation of 3$x_R$, while for a *w* = 2 $\mu$m target their energy is more than doubled.

Following the electrons over a long propagation distance using PIC is impractical because of heavy computational load, hence the low statistics of high-energy electrons in Figs. 3d, e. However, since the bunching of hot electrons observed in Fig. 3 indicates that the space charge forces between them are small, we are able to use a ”particle pusher” type simulation to study the single electron interaction with the diffracted laser beam over a long distance, in a parametric manner.

The transverse component of a Gaussian laser field is given by

1 $$\begin{aligned} E^{G}_y(y,x,t;w_0)=E_0\frac{w_0}{w} exp\bigg \{-y^{2}/w^{2} +i(kx-\textrm{arctan}(x/x_{R}) +ky^{2}/(2R_c) -\omega t)\bigg \} \end{aligned}$$

where the beam waist radius $w(x) = w_0 \sqrt{1 + \left( x/x_R\right) ^2}$ is set by its value at focus $w_0$ and its Rayleigh range $x_R=\pi w_0^2 / \lambda$, and where the radius of curvature is given by $R_c(x) = x + x_R^2/x$. The transverse and longitudinal fields of the diffracted beam, obscured by the micro-bar, are then given by^28^:

2 $$\begin{aligned} E_y=E^{G}_y(y,x,t;w_{beam})-E^{G}_y(y,x,t;w_{bar}),\qquad E_x=E_{y}\frac{y\cdot \textrm{cos}\big (\textrm{arctan}(y/x)\big )}{q_{0}+x} \end{aligned}$$

with $q_0 = \frac{i\pi \omega _0^2}{\lambda }$. The trajectories of electrons interacting with these fields having a set initial position and momentum at time t = 0, may be followed using a finite difference method:

3 $$\begin{aligned} \Delta \vec {p}=-e (\vec {E}+\vec {\beta }\times \vec {B})\Delta t \quad \rightarrow \quad \vec {p}(t+\Delta t)=\vec {p}+\Delta \vec {p} \quad \rightarrow \quad \vec {x}(t+\Delta t)=\vec {x}+\frac{\vec {p}}{\gamma m}\Delta t \end{aligned}$$

where $$\gamma =\sqrt{1+p^2/(mc^2)}$$ and $$\beta =|p|/\gamma m c$$.

**Figure 4:**
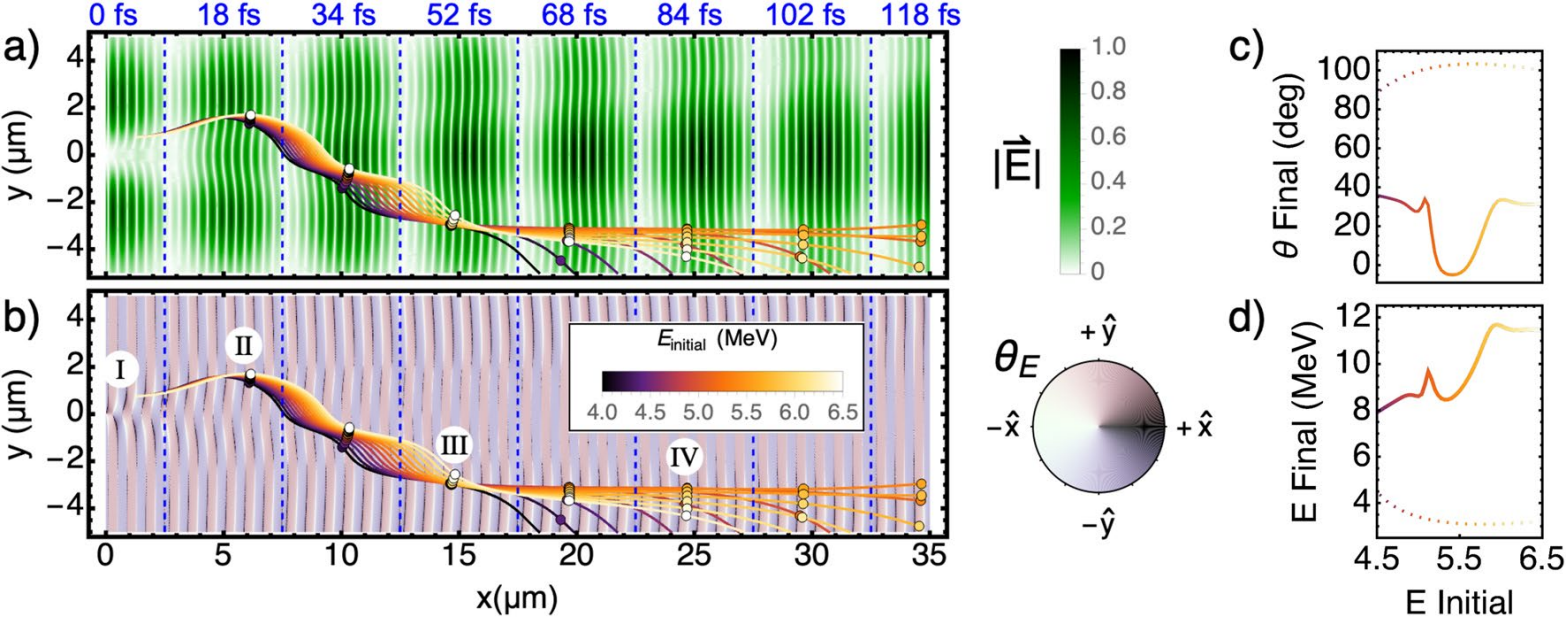
Simulation of electrons propagating in the diffracted laser field. Shown are eight snapshots of the electric field magnitude (**a**) and its direction (**b**), captured at equal intervals during the propagation of the pulse over one Rayleigh length ($x_R$ = 34.7 $\upmu$m). The trajectories of electrons, injected at ${\textcircled {{I}}}$ with initial energies in the range of 4–6.5 MeV, are overlaid in (a,b). The positions of the electrons at each time frame are marked with circles. (c) and (d) show the final emission angle and energy (solid curves), respectively, as a function of the initial electron energy. The color of the curve matches that of the trajectories in (a,b). Simulation results for electrons injected at the same initial position propagating under a Gaussian field (i.e. without a micro-bar) is indicated by dotted lines.

Figure 4 presents simulated trajectories of electrons interacting with the diffracted field in vacuum. The electric field’s magnitude (Fig. 4a) and direction (Fig. 4b) are plotted on eight temporal snapshots at equal intervals, starting at t = 0, where the peak intensity of the pulse is incident on a *w* = 2 $\upmu$m wide micro-bar positioned at $x = 0$. See Supplementary Video 2 for the full animation. The annular low-field channel formed in the diffracted field is clearly observed in Fig. 4a. The white slivers in Fig. 4b indicate regions in which an electron would be accelerated forward. These are the regions in which electrons are observed to be bunching in the PIC results (Fig. 3), therefore they served as the injection point (labeled ${\textcircled {{I}}}$) in this simulation.

The simulated trajectories of electrons initially moving forward with energies in the range of 4–6.5 MeV are overlaid on Fig. 4a, b. Their location at the time of each snapshot is indicated with a circle. While propagating forward, the electrons dephase with respect to the laser’s optical cycle according to their velocity. Between ${\textcircled {{I}}}$ and ${\textcircled {{II}}}$, the forward-pushing regions of the field are tilted with respect to *x*, so the electrons disperse transversely by different amounts according to the amount of their dephasing. At the shadow of the micro-bar ($-w/2< y < w/2$), the forward-pushing regions are observed to diminish as *x* approaches $x_R$, thus between ${\textcircled {{II}}}$ and ${\textcircled {{III}}}$ the electrons are expelled outward and slip back by two optical cycles, until they are trapped again in another white sliver, on the negative side of the *y* axis.

As *x* approaches $x_R$, the overall curvature of the diffracted field flattens. At ${\textcircled {{IV}}}$ only electrons of a narrow energy range, which settled at the center of the low field channel, are observed to be pushed forward, while slower or faster electrons are expelled by the transverse forces at the edges of the channel.

The final emission angle and energy are plotted in Fig. 4c, d as a function of the initial electron energy in a solid line with a color scale that matches their trajectories in Fig. 4a, b. The trapping of electrons of a narrow energy range, the overall net acceleration, and the positive angle-to-energy correlation that were observed in the experiment and PIC, are also featured here. Electron bunches injected with the same initial conditions into a Gaussian laser pulse unobscured by a micro-bar, are quickly expelled outward, as is observed by the dotted lines in Fig. 4c, d.

In conclusion, by irradiating single micrometric scale Au bars we revealed new features of how intense laser fields couple to wave-length scale objects. The physical process takes place in two steps: first, electrons that were pulled into vacuum near the edge of the target circumvent the cold material by the pull of the transverse electric field and the $v\times B$ motion under the laser’s magnetic field. This dynamics repeats at the peak of the field’s intensity every half-optical cycle, and results in a train of attosecond duration bunches of electrons. After passing the target, the diffracted laser fields interact with the electrons in vacuum over a distance of many Rayleigh ranges. The electrons bunch in small confined volumes in which the diffraction field pushes them forward, keeping them nearly in-phase with the laser pulse. But the structure of these volumes changes along the propagation direction, making this a highly selective process. The electrons that manage to stay in phase are accelerated and emitted in the form of two forward-directed jets ($\theta < 4^\circ$) comprised of attosecond duration bunches, while the rest are expelled by the transverse field. These emission characteristics motivate the use of such isolated micrometric target for generating short-wavelength radiation with attosecond pulse durations through Thomson scattering with a counter-propagating laser pulse or by seeding a free-electron laser^22^.

## Methods

### Target fabrication

The targets were free-standing Au bars suspended over rectangular openings in a 250 $\upmu$m thick Si wafer support. The fabrication process starts with a Si wafer pre-coated on its front with a 200-nm thick layer of high-stress Si$_3$N$_4$. The back side of the wafer is spin-coated with layers of resist (MicroChem SF9) and photoresist (MicroChem AZ-1518), on which 3.0 mm $\times$ 0.4 mm rectangular gaps are photolithographed. The Si is then etched in a 30% KOH solution at 90$^{\circ }$C. The process spontaneously stops when the inner surface of the front side Si$_3$N$_4$ is exposed. Next, the Si$_3$N$_4$ side of the wafer is spin-coated with layers of the same resist and photoresist. 2–6 $\upmu$m wide rectangular openings, which would form the micro-bars, are photolithographed over the gaps. The wafer is coated with a 10-nm thick Ti adhesion layer and a 190-nm thick layer of Au. The Si$_3$N$_4$ around the bars is removed by reactive ion etching and immersion in Acetone. Finally, the remaining Si$_3$N$_4$ layer below the Au bars is removed by dry-etching.

### PIC simulation

We used the fully relativistic EPOCH PIC code^25^ to carry out the simulations. One simulation was conducted in 3D, with minimal temporal and spatial resolutions and covering a brief duration, and four 2D simulations (with *w* = 1, 2, 3, and 4 $\upmu$m) to follow the trajectories of the emitted electrons over a significant distance in vacuum. In both cases, the laser pulse exhibited a Gaussian temporal profile with a width of 30 fs (FWHM) and a wavelength of 800 nm, at p-polarization. The laser beam was focused to a Gaussian intensity distribution with a radius of 3.5 $\upmu$m (FWHM), yielding peak laser intensity of $a_0$ = 4.6. The PIC simulations in Fig. 3d and e were performed with the actual laser intensity of the experiments, $a_0$ = 2.7.

The 3D simulation space was defined as a (6 $\upmu$m)$^3$ box divided into (100)$^3$ computational mesh cells. The 2D simulation space was defined as a (18 $\upmu$m)$_{\bot } \times$ (30 $\upmu$m)$_{\parallel }$ box with $(6144)_{\bot } \times (6144)_{\parallel }$ computational mesh cells. Here $\bot$ and $\parallel$ are with respect to the laser propagation direction. The 2D simulations ran for 40 fs, after which the simulation box was set to move in the laser propagation direction at the speed of light, for additional 350 fs.

The targets were representative of electrons, protons, and singly-ionized Au ions. Their initial distribution was rectangular, of (2–6 $\upmu$m)$_\bot$ $\times$ (0.2 $\upmu$m)$_\parallel$, with a uniform density of 3000 times the plasma’s critical density, and a density exponential gradient around them with a scale length of 13.3-nm ($\lambda$ / 60). For the 3D simulation, this density profile was extruded over the third direction to 10 $\upmu$m.

### Experimental setup

**Figure 5:**
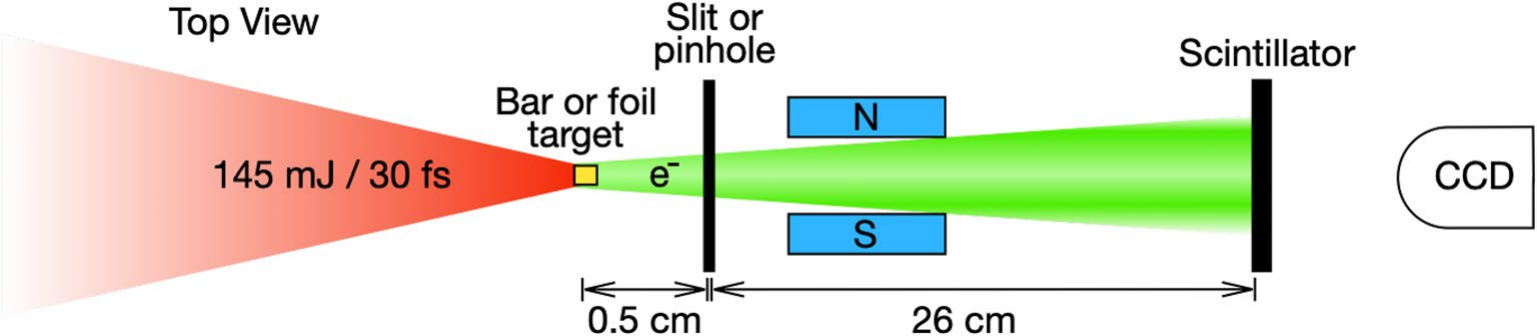
Top-view cartoon drawing of the experimental setup (elements are not to-scale). The long dimension of the micro-bar is out-of-plane. See text for details.

We performed the experiments using the NePTUN 20 TW laser system at Tel Aviv University^29^. Figure 5 shows cartoon schematics of the experimental setup. 30-fs long laser pulses with energies of 145 mJ (on-target) and pulse contrast better than $10^{11}$^30^, were focused using an f/2.5 off-axis parabolic mirror unto the Au bar targets. We measured 70% of the laser energy to be contained within a circle of 3.5 $\upmu$m diameter.

We recorded electron spectra using a charge-coupled device imaging a CsI(Tl) scintillator^31^ at the focal plane of magnetic spectrometers. We used two configurations to generate the spectrograms shown in Figs. 1 and 2: (1) An acceptance of $\Omega$ = 0.12 msr and 0.2 < E$_e<$ 4.2 MeV, as described in detail in the Supp. note of Ref.^32^, but with a reduced magnetic field of 0.25 T. The scintillator was covered on its target-facing side with a 15-$\upmu$m thick aluminum foil to block scattered light. (2) A wide angular acceptance spectrometer of $\theta$ = 182 mrad and 0.9 < E$_e<$ 6 MeV, which consisted of a 1.7 cm (horizontal) $\times$ 400 $\upmu$m (vertical) wide slit followed by a 0.5 cm long 0.12 T magnetic field, positioned 3.5 cm downstream from the target. A 15 cm $\times$ 10 cm $\times$ 0.8 cm scintillator was placed 26 cm downstream from the slit and was coated with 600-nm thick layer of aluminum to block scatter light.

### Particle pusher simulation

**Figure 6:**
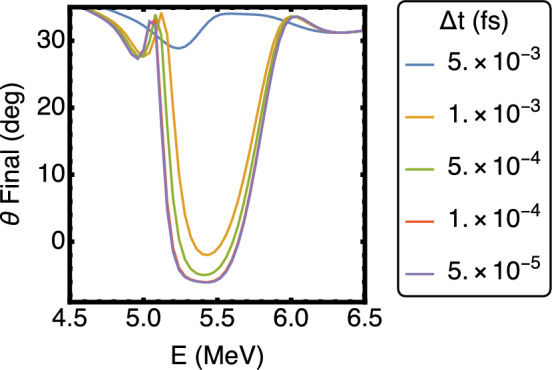
The final emission angles of electrons simulated with the Particle Pusher simulation code. Numerical stability of the code is achieved for $\Delta$ t $<5\times$ 10$^{-4}$ fs.

The code simulated the trajectories of electrons by iterating over Eq. 3, where the laser’s electric field was calculated using Eqs. 2, and where its out-of-plane magnetic field was given by $B_z=\frac{1}{k}(\frac{dE_y}{dx}-\frac{dE_x}{dy})$. In calculating the fields, the laser wavelength, the waist of the incoming Gaussian beam, and the width of the micro-bar were taken to be: $\lambda$ = 0.8 $\upmu$m, $w_{beam}$ = 3.5 $\upmu$m (FWHM), and $w_{bar}$ = 2 $\upmu$m. The numerical stability of the code was confirmed by inspecting the electron trajectory dependence on $\Delta$t. Figure 6 shows the final emission angles of electrons that were simulated on the same conditions as in Fig. 4. The curves show how the integration result stabilizes for $\Delta$t $<5\times$10$^{-4}$ fs, which is the value chosen for the analysis presented in this paper.

### Supplementary Information

Supplementary Legends.

Supplementary Video 1.

Supplementary Video 2.

## Data Availability

The datasets used and/or analysed during the current study available from the corresponding author on reasonable request.
