# Supplementary material for: Intense laser interaction with micro-bars
Source: Sci Rep. 2023 Dec 4;13:21345. doi: 10.1038/s41598-023-48866-z (PMC10696094; doi:10.1038/s41598-023-48866-z)
Supplement: Supplementary file 1 — Supplementary Legends. [file 41598_2023_48866_MOESM1_ESM.docx]

Supplementary video 1:

Results of a 2D-PIC simulations for the irradiation of a 0.2 μm thick, 1.0 μm wide micro-bar. The transverse component of the electric field (Ey) is shown in a red-to-blue color scale. The density of electrons having energy above a threshold of 1 MeV is shown in green. See the Methods section for a complete technical description of the simulated conditions.

Supplementary video 2:

Simulation of electrons propagating in the diffracted laser field, over a distance of one Rayleigh length. The electric field direction is plotted in white-to-magenta-to-black scale (see legend in Fig. 4). The trajectories of electrons, injected at the same initial point with initial energies in the range of 4–6.5 MeV, are indicated by the color-coded curves.
